# Supplementary material for: Providers’ mediating role for medication adherence among cancer survivors
Source: PLoS One. 2021 Nov 29;16(11):e0260358. doi: 10.1371/journal.pone.0260358 (PMC8629272; doi:10.1371/journal.pone.0260358)

**S1 Appendix**

**CAUSAL MODEL**

Our causal model is based on Bind et al.^1^ (**Figure 1**). Cancer, the primary exposure, causes changes to a survivor’s provider team (e.g., addition of oncologist), directly affects medication adherence for chronic conditions (e.g., increasing pill burden, introducing competing goals, or serving as a “wake up” call to improve health), and persists into the future. The provider team affects medication adherence through monitoring and coordination of treatment regimens. The provider team and adherence are influenced by survivor characteristics, observed ($X_{i}$) and unobserved ($\mu_{i}$).

Our estimation approach relies on within-person variation in cancer status, provider team structure and medication adherence. Equations (1) and (2) represent the causal model:

$E\left[ P_{it}^{k} | X_{i}, C_{it}, \mu_{i} \right]=\beta_{0}^{k}+\beta_{1}^{k}C_{it}+\beta_{x}^{k}X_{i}+\mu_{i}$ (1)

$E[Y_{it}|X_{i}, C_{it}, \boldsymbol{P}_{it},\theta_{i}]=\gamma_{0}+\gamma_{1}C_{it}+\sum_{k} \gamma_{2}^{k}P_{it}^{k}+\gamma_{x}X_{i}+\theta_{i}$ (2)

where $P_{it}^{k}$ is a measure (*k*) of the provider team (of *K* measures) for survivor *i* in time *t*. Provider team measures represent features of the survivor’s care team such as the number of total providers, number of specialists, and extent of historical patient sharing among the survivor’s providers (a proxy for opportunities for care coordination).^2-7^ $X_{i}$ are observable, time-invariant survivor characteristics (e.g., age at diagnosis, sex, race, SEER region, stage at diagnosis); $C_{it}$ is cancer status (yes/no); $\mu_{i}$ is unobserved, time-invariant survivor characteristics influencing the choice of provider team (e.g., care-seeking behavior); $Y_{it}$ is chronic condition medication adherence; and $\theta_{i}$ is unobserved, time-invariant survivor characteristics influencing medication adherence (e.g., health literacy).

**Identifying assumptions**

Following Bind et al.,^1^ we also made important identifying assumptions for dynamic relationships. First, for all *t* = 1 to *T*-1 and for all *t’* > *t*, $P_{it}$ and $Y_{it}$ do not affect $C_{it^{'}}$. Thus, we assume the provider team does not affect cancer status next period, for example through better screening. However, a relationship between provider team and cancer status *is* allowed within the period, which was six months. The provider team can also be correlated with next period’s cancer status through the unobserved survivor characteristics ($\mu_{i}$). Second, we assume that $C_{it}$ and $Y_{it}$ do not affect $P_{it^{'}}$ (e.g., cancer status does not affect provider team next period). Thus, any changes to the provider team due to cancer happen *within* a six-month period. In addition, our model allows correlation between cancer status and the provider team next period through the unobserved survivor characteristics ($\mu_{i}$).

In addition, we assume no unobserved confounding in any of the regressions from equations (1) and (2) conditional on $X_{i}$, $\theta_{i}$, and $\mu_{i}$. Specifically, let $Y_{it}^{c,p}$represent potential adherence for person *i* in time *t* with cancer status set to *c* and the provider team set to *p*.

$$Y_{it}^{c,p}\coprod C_{it}|X_{i}, \mu_{i},\theta_{i}$$

No unmeasured cancer-adherence confounding at time *t* given the covariates and fixed effects.

$$Y_{it}^{c,p}\coprod P_{it}|C_{it},X_{i}, \mu_{i},\theta_{i}$$

No unmeasured provider team-adherence confounding given cancer status, the covariates and fixed effects.

$$P_{it}^{c}\coprod C_{it}|X_{i}, \mu_{i},\theta_{i}$$

No unmeasured cancer-provider team confounding given the covariates and fixed effects.

$$Y_{it}^{c,p}\coprod P_{it}^{c^{*}}|X_{i}, \mu_{i},\theta_{i}$$

No unmeasured provider team-adherence confounder affected by cancer status.

Our specification allows unobserved, contemporaneous confounding between cancer status and provider team (i.e., $\mu_{i}$ can be correlated with $C_{it}$ in the *K* separate regressions for equation (1)) and between cancer status, provider team and adherence (i.e., $\theta_{i}$ can be correlated with $C_{it}$ and $P_{it}^{k}$ in equation (2)). In addition, $\mu_{i}$ and $\theta_{i}$ can be correlated in an unrestricted way; **Figure 1** assumes the two unobserved terms are identical ($\theta_{i}=\mu_{i}$).

**Natural Direct Effect (cancer on adherence)**

NDE is the expected difference in adherence between those with and without cancer holding the care team constant in the non-cancer configuration.

$$NDE=E[Y_{it}^{c,p_{it}^{c^{*}}}-Y_{it}^{c^{*},p_{it}^{c^{*}}}|X_{i}=x,\mu_{i},\theta_{i}]$$

$$=\gamma_{0}+\gamma_{1}c+\sum_{k} \gamma_{2}^{k}(\beta_{0}^{k}+\beta_{1}^{k}c^{*}+\beta_{x}^{k}X_{i}+\mu_{i})+\gamma_{x}X_{i}+\theta_{i}$$

$$-\left( \gamma_{0}+\gamma_{1}c^{*}+\sum_{k} \gamma_{2}^{k}(\beta_{0}^{k}+\beta_{1}^{k}c^{*}+\beta_{x}^{k}X_{i}+\mu_{i})+\gamma_{x}X_{i}+\theta_{i} \right)$$

$$=\gamma_{1}\left( c-c^{*} \right)$$

Let *c* = 1 and *c^*^* = 0. Then NDE = $\gamma_{1}$.

**Natural Indirect Effect (cancer -> provider team -> adherence)**

NIE is the expected difference in adherence among cancer patients comparing care teams with and without cancer.

$$NIE=E[Y_{it}^{c,p_{it}^{c}}-Y_{it}^{c,p_{it}^{c^{*}}}|X_{i}=x,\mu_{i},\theta_{i}]$$

$$=\gamma_{0}+\gamma_{1}c+\sum_{k} \gamma_{2}^{k}(\beta_{0}^{k}+\beta_{1}^{k}c+\beta_{x}^{k}X_{i}+\mu_{i})+\gamma_{x}X_{i}+\theta_{i}$$

$$-\left( \gamma_{0}+\gamma_{1}c+\sum_{k} \gamma_{2}^{k}(\beta_{0}^{k}+\beta_{1}^{k}c^{*}+\beta_{x}^{k}X_{i}+\mu_{i})+\gamma_{x}X_{i}+\theta_{i} \right)$$

$$=\sum_{k} \gamma_{2}^{k}\beta_{1}^{k}\left( c-c^{*} \right)$$

Let *c* = 1 and *c^*^* = 0. Then NIE = $\sum_{k} \gamma_{2}^{k}\beta_{1}^{k}$.

**References**

1. Bind MA, Vanderweele TJ, Coull BA, Schwartz JD. Causal mediation analysis for longitudinal data with exogenous exposure. *Biostatistics.* 2016;17(1):122-134.
2. Pollack CE, Frick KD, Herbert RJ, et al. It's who you know: patient-sharing, quality, and costs of cancer survivorship care. Journal of cancer survivorship : research and practice. 2014;8(2):156-166.
3. Trogdon JG, Chang Y, Shai S, et al. Care coordination and multispecialty teams in the care of colorectal cancer patients. Medical care. 2018;56(5):430-435.
4. Barnett ML, Landon BE, O'Malley AJ, Keating NL, Christakis NA. Mapping physician networks with self-reported and administrative data. Health services research. 2011;46(5):1592-1609.
5. Landon BE, Onnela JP, Keating NL, et al. Using administrative data to identify naturally occurring networks of physicians. Medical care. 2013;51(8):715-721.
6. Pollack CE, Weissman G, Bekelman J, Liao K, Armstrong K. Physician social networks and variation in prostate cancer treatment in three cities. Health services research. 2012;47(1 Pt 2):380-403.
7. Trogdon JG, Weir WH, Shai S, et al. Comparing Shared Patient Networks Across Payers. Journal of general internal medicine. 2019;34(10):2014-2020.

**Figure 1. Causal model for the role of cancer in medication adherence for chronic conditions.** *Cancer_it_* = cancer status of person *i* at time *t* (exposure); *Providers_it_* = provider team for person *i* at time *t* (mediator); *Adherence_it_* = chronic condition medication adherence for person *i* at time *t* (outcome); *X_i_* = observed patient characteristics for person *i* (confounder); and *U_i_* = unobserved patient characteristics for person *i* (confounder).


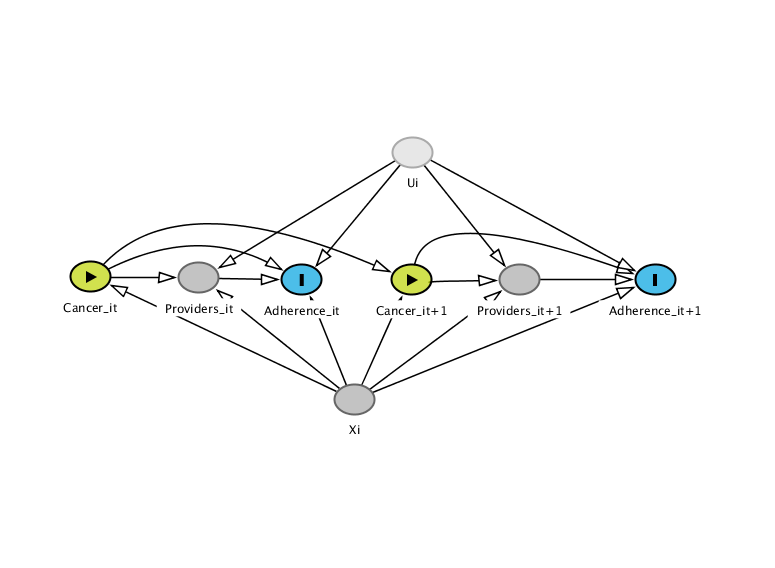

Supplement: S1 Appendix — (DOCX) [file pone.0260358.s001.docx]
