# Supplementary material for: Providers’ mediating role for medication adherence among cancer survivors
Source: PLoS One. 2021 Nov 29;16(11):e0260358. doi: 10.1371/journal.pone.0260358 (PMC8629272; doi:10.1371/journal.pone.0260358)

**S1 Figure**

**Figure 1A-C**. **Total effect of cancer on discontinuation by chronic condition cohort, cancer site, and phase of care.** Point estimates and 95% confidence intervals.

(A) Non-insulin anti-diabetics


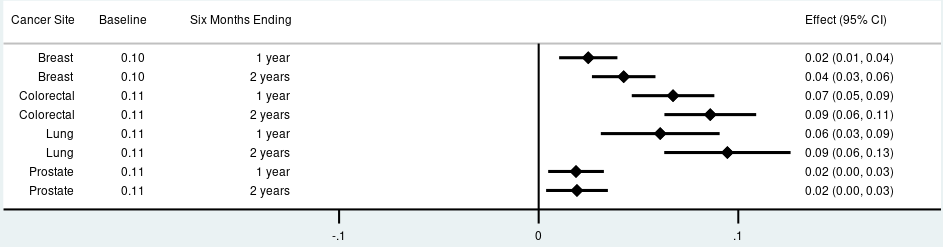


(B) Statins


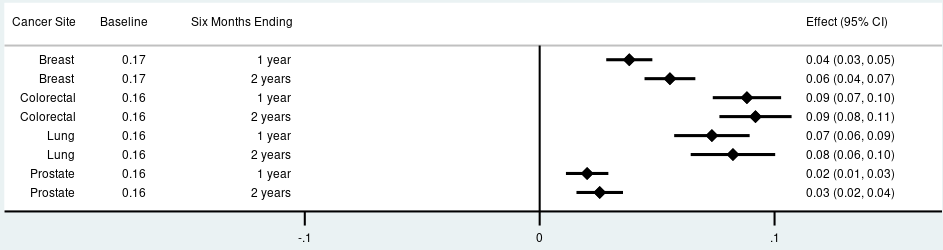


(C) Anti-hypertensives


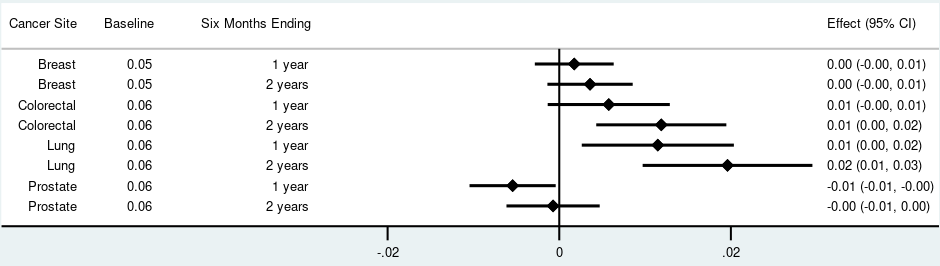


**Figure 2A-C. Effects of cancer on discontinuation: total effect (TE), natural direct effect (NDE) and natural indirect effect (NIE) through number of providers and sharing amongst providers.** Point estimates, in percentage point changes, for chronic condition cohorts, cancer sites, and phases of care with statistically significant total and net indirect effect: non-insulin anti-diabetics (A), statins (B), and anti-hypertensives (C).

(A) Non-insulin anti-diabetics

**
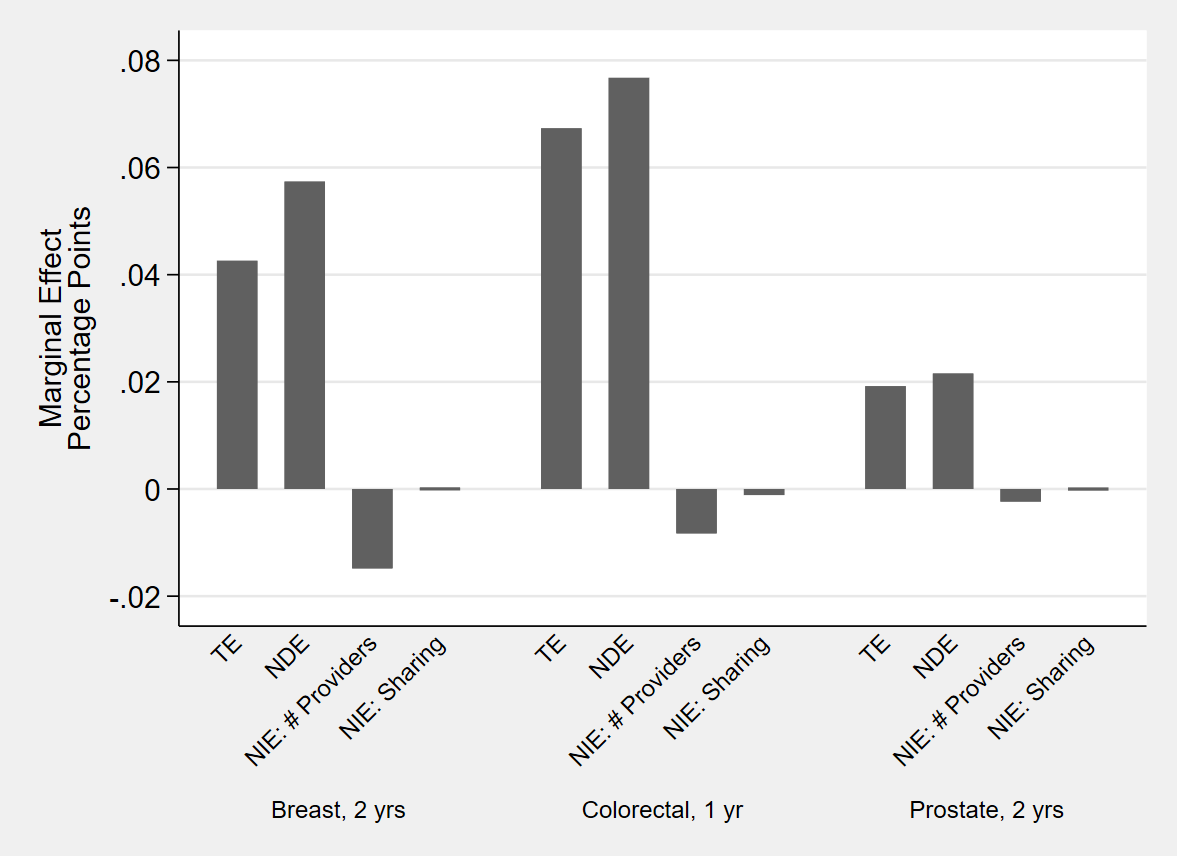
**

(B) Statins


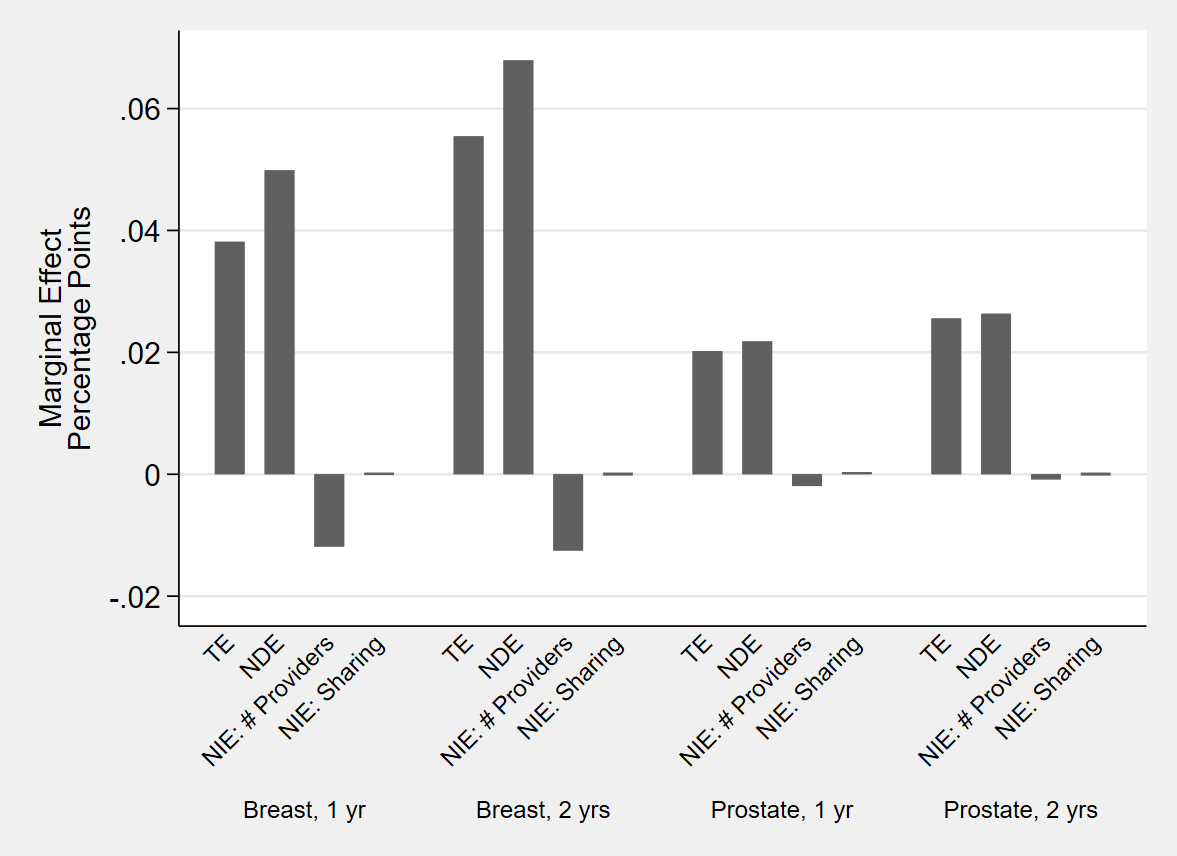


(C) Anti-hypertensives


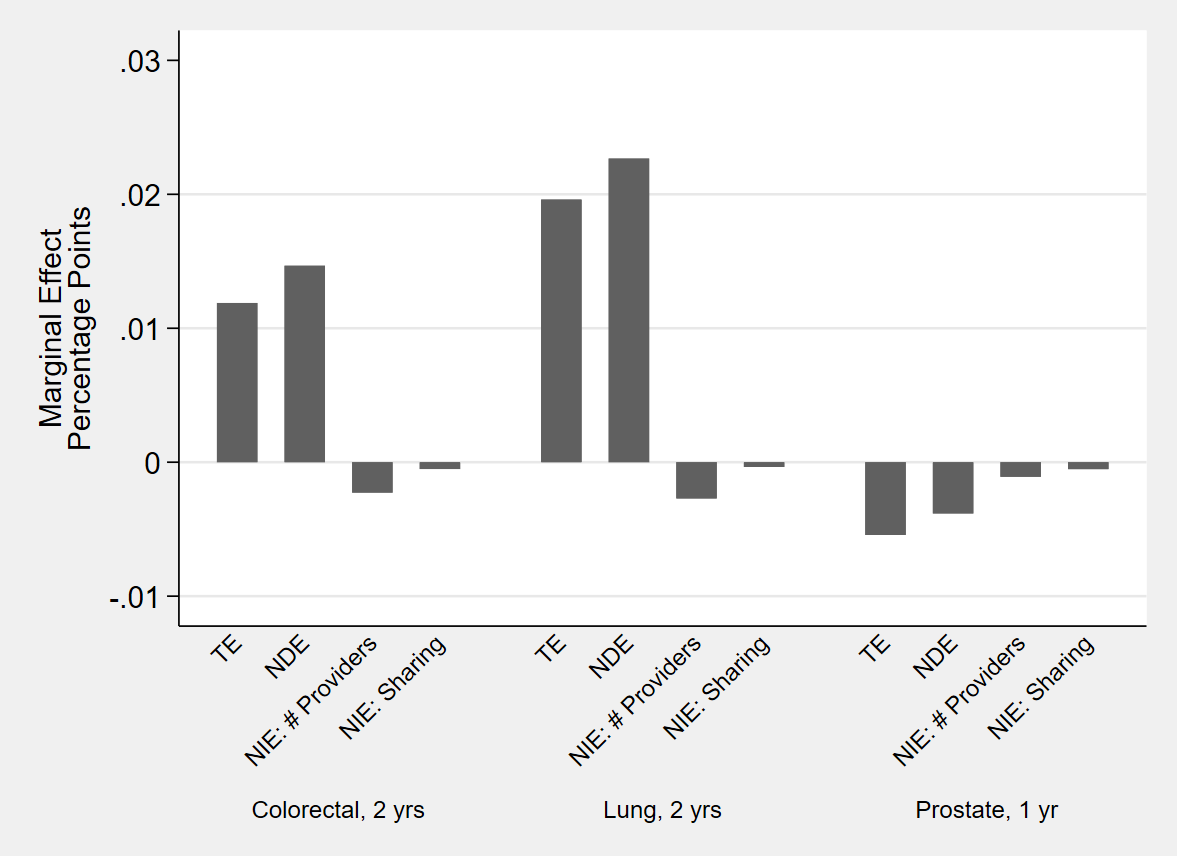

Supplement: S1 Fig — (DOCX) [file pone.0260358.s002.docx]
