# Supplementary material for: Providers’ mediating role for medication adherence among cancer survivors
Source: PLoS One. 2021 Nov 29;16(11):e0260358. doi: 10.1371/journal.pone.0260358 (PMC8629272; doi:10.1371/journal.pone.0260358)
Supplement: S1 Table — (DOCX) [file pone.0260358.s003.docx]

**S1 Table. Factor weights by cancer site and chronic condition.**

|  |  |  | Breast |  | Colorectal |  | Lung |  | Prostate |  |
| --- | --- | --- | --- | --- | --- | --- | --- | --- | --- | --- |
|  |  |  | Factor | | Factor | | Factor | | Factor | |
| Chronic Condition | Time | Provider Team Variable | Number | Sharing | Number | Sharing | Number | Sharing | Number | Sharing |
| Diabetes | 1 year | Providers | 0.738 | -0.093 | 0.710 | -0.084 | 0.683 | -0.073 | 0.683 | -0.048 |
|  |  | Specialists | 0.766 | 0.006 | 0.719 | 0.002 | 0.696 | 0.025 | 0.693 | 0.011 |
|  |  | Shared Patient Volume | -0.013 | 0.243 | -0.019 | 0.267 | -0.062 | 0.225 | -0.050 | 0.191 |
|  |  | Degree | 0.387 | 0.174 | 0.324 | 0.197 | 0.319 | 0.145 | 0.207 | 0.165 |
|  | 2 years | Providers | 0.695 | -0.078 | 0.700 | -0.070 | 0.685 | -0.081 | 0.668 | -0.058 |
|  |  | Specialists | 0.715 | 0.015 | 0.708 | 0.006 | 0.700 | 0.019 | 0.674 | 0.009 |
|  |  | Shared Patient Volume | -0.026 | 0.224 | -0.009 | 0.230 | -0.092 | 0.277 | -0.030 | 0.237 |
|  |  | Degree | 0.297 | 0.166 | 0.279 | 0.167 | 0.290 | 0.234 | 0.195 | 0.205 |
|  |  | N | 7,888 |  | 4,932 |  | 2,496 |  | 8,548 |  |
| Statins | 1 year | Providers | 0.766 | -0.105 | 0.711 | -0.094 | 0.698 | -0.090 | 0.690 | -0.061 |
|  |  | Specialists | 0.787 | -0.023 | 0.726 | -0.016 | 0.721 | 0.010 | 0.698 | 0.007 |
|  |  | Shared Patient Volume | 0.081 | 0.264 | 0.013 | 0.288 | 0.011 | 0.238 | -0.016 | 0.248 |
|  |  | Degree | 0.405 | 0.191 | 0.328 | 0.229 | 0.328 | 0.162 | 0.186 | 0.221 |
|  | 2 years | Providers | 0.730 | -0.098 | 0.696 | -0.078 | 0.696 | -0.084 | 0.682 | -0.065 |
|  |  | Specialists | 0.756 | -0.004 | 0.712 | 0.005 | 0.720 | 0.012 | 0.689 | -0.004 |
|  |  | Shared Patient Volume | 0.047 | 0.248 | -0.013 | 0.251 | -0.035 | 0.242 | 0.008 | 0.263 |
|  |  | Degree | 0.326 | 0.194 | 0.252 | 0.213 | 0.290 | 0.201 | 0.193 | 0.235 |
|  |  | N | 25,896 |  | 14,236 |  | 9,820 |  | 29,104 |  |
| Anti-hypertensives | 1 year | Providers | 0.758 | -0.102 | 0.718 | -0.084 | 0.704 | -0.076 | 0.687 | -0.058 |
|  |  | Specialists | 0.786 | -0.020 | 0.732 | -0.015 | 0.721 | -0.004 | 0.697 | 0.004 |
|  |  | Shared Patient Volume | 0.071 | 0.250 | 0.008 | 0.267 | -0.012 | 0.242 | -0.004 | 0.240 |
|  |  | Degree | 0.400 | 0.187 | 0.333 | 0.208 | 0.310 | 0.190 | 0.172 | 0.223 |
|  | 2 years | Providers | 0.726 | -0.090 | 0.705 | -0.077 | 0.702 | -0.080 | 0.680 | -0.069 |
|  |  | Specialists | 0.755 | -0.003 | 0.723 | 0.005 | 0.722 | 0.005 | 0.688 | 0.002 |
|  |  | Shared Patient Volume | 0.029 | 0.234 | -0.013 | 0.244 | -0.038 | 0.254 | 0.002 | 0.257 |
|  |  | Degree | 0.323 | 0.189 | 0.263 | 0.205 | 0.301 | 0.208 | 0.202 | 0.224 |
|  |  | N | 41,228 |  | 22,880 |  | 14,280 |  | 39,040 |  |
