# Supplementary material for: Providers’ mediating role for medication adherence among cancer survivors
Source: PLoS One. 2021 Nov 29;16(11):e0260358. doi: 10.1371/journal.pone.0260358 (PMC8629272; doi:10.1371/journal.pone.0260358)
Supplement: Attachment — Submitted filename: PLOS_One_response_reviewers_final_v2.docx [file pone.0260358.s004.docx]

*We thank the reviewers for their careful and thoughtful questions and suggestions. We have incorporated these comments in our revised paper and believe the paper has improved as a result. Our responses to each specific comment are below in italics.*

**Reviewer(s)' Comments:**

Reviewer 1

1. It is understandable that the authors have focused on 4 most common cancers, but it is not clear why in each set of cancers they have limited their population. For example for breast cancer, why stage IV was not included? or in lung cancer, why small cell lung was not included. If there is concern about heterogeneity in management, similar heterogeneity can be expected with colorectal cancer stages as well.

*The reviewer is correct that the main motive for selecting the cancer populations we did was to maximize generalizability by selecting the most common cancers, while also limiting heterogeneity in management within these cancer sites (e.g., excluding small cell lung cancer). We excluded stage IV and metastatic disease for all cancer sites due to the different incentives patients and providers would face for maintenance of chronic condition medications considering limited life expectancy. Our statistical analysis relied on within patient variation over time, which controls for the potential confounding effects of tumor features at diagnosis (e.g., stage, hormone receptor status for breast cancer).*

*We added justification for excluding stage IV/metastatic disease on p. 7: “We excluded stage IV and metastatic disease for all cancer sites due to the different incentives patients and providers would face for chronic condition medication adherence considering limited life expectancy.”*

*We also added tumor characteristics to the examples of variables that our approach adjusts for on p. 10: “**All regression variables were expressed as deviations from individual means over time to eliminate time-invariant patient characteristics (e.g., tumor features at diagnosis).” And p. 17: “**Our estimates also adjust for other factors influencing adherence that do not change over time (e.g., “healthy users,” health literacy, tumor stage).”*

*Finally, we added the exclusion of stage IV/metastatic disease to the limitations (p. 17): “Second, this study used data from 2008-2014 and was restricted to adults age 66 and older with continuous Medicare fee-for-service and Part D coverage* *with non-metastatic cancer who also survived two years following their cancer diagnosis. As such, our findings may not be generalizable to more current patients, those with Medicare Advantage or without prescription drug or other healthcare insurance, the population 65 years and under,* *patients with metastatic disease, or patients with a short life expectancy.”*

2. One of the main limitation of the study is that the data is from a decade ago, and might not be generalizable to current practices.

*We added the age of the data as a limitation in the Discussion (p. 17). See last statement in response to #1 above.*

3. In assigning provider team structure, there has been no mention of mid levels who commonly manage patients as primary care. Why mid levels were excluded from the study?

*We added the following justification to the paper (p. 9): “We excluded mid-level providers (e.g., nurse practitioners) due to the variety of regulations on prescriptive authority across states.”*

4. It is not clear why patient sharing with other providers, or count of all providers sharing patients (degree) is increasing among cancer patients vs. controls (in Table 1). Why a single patient cancer diagnosis impacting the patient volume shared?

*We added the following explanation to the Results (p. 11): “The increase in patient volume shared among cancer patients is due to higher rates of patient sharing among oncologists, who often become cancer patients’ main provider.”*

5. Authors may want to expand on why they think there is differences in adherence based on the chronic condition (why adherence to anti hypertensives higher) or type of cancer. Also there are controversial publications in this area; for example some other papers have shown breast cancer patients will have decreased adherence to chronic medications (as opposed to this study).

*We added the following to the Discussion to expand on why differences in our adherence results might exist across condition and cancer type (pp. 17-18):*

*“The decreases in adherence among colorectal and lung cancer patients are consistent with a hypothesis that patients diagnosed with more deadly cancer have decreased incentive to manage chronic conditions, perhaps because preventive medication has a long lag-time for benefit^47^ or because more complex treatments (e.g., surgical resection and adjuvant chemotherapy) change the need or benefits of continued use of chronic disease medications.^13^ Conversely, patients diagnosed with cancers with high survival probabilities (e.g., breast and prostate) are motivated to improve their (secondary) prevention efforts, including medication adherence. The positive effects of cancer diagnosis on antihypertensive medication adherence may be explained by the fact that blood pressure monitoring is routine during cancer care visits, which provides ample opportunity for providers to promote the importance of antihypertensive adherence. Alternatively, monitoring of lipid and blood sugar levels is less routine, which might partially explain the decreases in adherence to these medications among cancer patients. Of course, other explanations are also plausible.”*

6. In supplemental appendix, there is an error displaying Fig 2A-C, and the images could not be visualized.

*We apologize for this inconvenience. We made sure the images met PLOS requirements for submission.*

Reviewer 2

1. Was there censorship due to death? That is to say, that those with poorly coordinated care might have shorter survival. I think not, given that the limitations suggest that only those surviving two years are excluded. I think this is a point worth extracting out a bit: that it’s possible that provider complexity may have been important to survival overall, but, by design, that was not assessed in this analysis.

*Our analysis excluded patients who died within two years of (pseudo) diagnosis and acknowledged that our results would not generalize to those with a shorter life expectancy. Additionally, we added the following to the limitations (p. 17): “**Furthermore, we were not able to assess the effect of care coordination on mortality.”*

2. I’m not familiar with the term “demeaning”. Is this similar to group mean centering or grand mean centering?

*We apologize for the unclear language. Yes, our process was similar to group mean centering where the group mean is equal to each patient’s mean over time. We updated the language to better describe our process (p. 10): “**We transformed the four provider team measures to be deviations from individual means by subtracting each patient’s mean over time from each variable (i.e.,* $X_{it}^{new} = X_{it} - \bar{X}_{i.}$*).” And p. 10: “All regression variables were expressed as deviations from individual means over time to eliminate time-invariant patient characteristics (e.g., tumor features at diagnosis).”*

3. It seems that this analysis has time nested in patients nested in providers. Were hierarchical models used? If no hierarchical models were used, how would you account for shared variance due to clustering of patients within providers?

*Yes, our data has time nested in patients nested in providers. However, rather than use hierarchical models that rely on variation within* ***and between*** *patients and providers, we used the “within transformation,” described above, to identify our effects of interest using only variation within patients over time. The benefit of this approach is that time invariant observed differences between higher level units (i.e., providers) do not bias our estimates.*

4. Some of the cancers include long-term therapies that might also compete with comorbidity management. For example, endocrine therapy for breast cancer may be last up to 5 years, and include both oral and intravenous components. Were those medications also accounted for in some way?

*We did not account for long-term cancer therapies explicitly in our analysis. The effects of long-term therapies like endocrine therapy will contribute to the estimates of the net direct effect (NDE) of cancer diagnosis in our analysis. As described above and on pp. 17-18, such treatments could compete with chronic disease medication adherence and be part of the explanation for the decreases in adherence to these drugs for some cancers. We expanded our limitations in the Discussion to acknowledge this (p. 17): “**Fourth, several mechanisms exist for the NDE of cancer (e.g., competing long-term cancer therapies like endocrine therapy) and more research is needed to unpack these mechanisms.”*

5. The mediation model adds value, but I also wonder if there are moderating effects. Given the results in the figures showing differences by tumor site, have you explored whether the interaction of provider complexity with tumor site might lead to differences in adherence?

*We agree with the reviewer that moderating effects are of interest. The reviewer can view the stratified analysis by cancer site as allowing flexibly for cancer site (and chronic disease) to moderate the effect of provider complexity on adherence. The NIE results suggest small, if any, differences in the effects of provider complexity across cancer site and chronic conditions.*

6. Some of the language in the discussion extends beyond what the results of this study suggest, especially with the language about the need to focus on patient-level factors. Currently, it reads as if patient self-efficacy is the culprit for adherence challenges, as if the problem is that patients just aren’t motivated enough to be adherent. It comes across as victim-blaming and as if increasing a sense of personal responsibility will fix adherence issues. Current interventions for medication adherence for cancer survivors that focus on patient-level factors suggest otherwise1-5. If we can reasonably assume that patients do care about their health to some degree, there are likely competing priorities that may be wholly outside of health care that patients are busy addressing and influences their adherence. While this paper providers strong evidence about complexity of the provider team, there may be other provider-level factors that are still important that are not explored here. It is premature to suggest we should switch to focusing on patient factors. I recommend this language on p14 be revised substantially.

*We appreciate the reviewer’s comments regarding the focus on patient-level factors and did not intend to blame the patient. As suggested, we have substantially revised the Discussion to remove any blame pointing to the patient and to highlight other provider- and area-level factors that could be addressed (p. 16):*

*“While this paper provides evidence about complexity of the provider team, several other factors may influence adherence to chronic medications including other provider characteristics (e.g., communication, reimbursement arrangements), social determinants of health, the financial burden of cancer treatment, and physiological responses to cancer and its treatments. Social determinants of health are the conditions in the environments where people live (e.g., transportation, stable housing) and have been linked to medication adherence.^42^ The financial toxicity of cancer treatment is a known constraint for many patients,^43,44^ which may translate beyond cancer into the management of comorbid chronic conditions. Additionally, the chemotherapeutic toxicity of many cancer treatments (e.g., emetogenic chemotherapy) can reduce a patient’s adherence to comorbid medications.^45^ Furthermore, physiological responses may result in imbalances in blood pressure and blood glucose control, which can influence medication adherence.^8,46^ It is important to understand the range of factors that may influence chronic condition medication adherence as the interventions to improve each of these factors vary significantly.”*

7. One of the values of this paper is that it gives us a start on the evidence around the influence of providers: but also leaves more room to explore. For example, might provider complexity influence disparities in adherence? Might there be rural-urban differences to consider or issues of geography? Rather than saying we should immediately shift to patient-level factors, it would be worthwhile to point to other provider-level issues to explore.

*We now emphasize other provider- and area-level issues in our response to comment #6.*

**Journal Requirements**

1. Please provide additional information about the patient records used in your retrospective study, including the date range (month and year) during which patients' medical records were accessed

*We revised the Data source and study populations section of the Methods to include the requested date ranges (p. 7): “We identified patients aged ≥66 with a first primary diagnosis of stage I-III breast, prostate, non-small cell lung, or colorectal cancer from July 1, 2008 – December 31, 2012. Access to the data was originally granted in 2017.”*

2. In your revised cover letter, please address the following prompts:

a) If there are ethical or legal restrictions on sharing a de-identified data set, please explain them in detail (e.g., data contain potentially identifying or sensitive patient information) and who has imposed them (e.g., an ethics committee). Please also provide contact information for a data access committee, ethics committee, or other institutional body to which data requests may be sent.

*Our cover letter now includes the following revised statement: “The SEER-Medicare data is owned by the SEER registry Principal Investigators and the Centers for Medicare and Medicaid Services. Although personal identifiers for all patient and medical care providers have been removed from the SEER-Medicare data, there remains the remote risk of re-identification (given the large amount of data available). Data can be accessed, subject to approval and data use agreement, from the Healthcare Delivery Research Program at the National Cancer Institute (*[*http://appliedresearch.cancer.gov/seermedicare/obtain/requests.html*](http://appliedresearch.cancer.gov/seermedicare/obtain/requests.html)*).”*

3. We note that the grant information you provided in the ‘Funding Information’ and ‘Financial Disclosure’ sections do not match. When you resubmit, please ensure that you provide the correct grant numbers for the awards you received for your study in the ‘Funding Information’ section.

*Below is the correct information for the funding we received for the study: “This research was support by the National Institute on Aging (NIA R01 AG050733; PI: Trogdon; https://www.nia.nih.gov/). The database infrastructure used for this project was supported through the University of North Carolina Clinical and Translational Science Award (UL1TR001111) and the UNC Lineberger Comprehensive Cancer Center, University Cancer Research Fund via the State of North Carolina. The funders had no role in study design, data collection and analysis, decision to publish, or preparation of the manuscript.”*

*We list as Competing Interests other relevant funding outside of this study.*

4. Please include your updated Competing Interests statement in your cover letter. Please confirm that this does not alter your adherence to all PLOS ONE policies on sharing data and materials, by including the following statement: "This does not alter our adherence to PLOS ONE policies on sharing data and materials.” If there are restrictions on sharing of data and/or materials, please state these.

*We updated our Competing Interests statement as requested:*

*“Dr. Lund’s spouse is a full-time, paid employee of GlaxoSmithKline who also holds stock in the amount of approximately $42,000. Dr. Lund also receives unrelated grant funding paid to her institution from AbbVie. Drs. Reeder-Hayes and Wheeler receive unrelated grant funding paid to their institution from Pfizer. Dr. Farley received unrelated grant funding paid to his institution from Astra Zeneca. All other co-authors have no potential competing interests to report. This does not alter our adherence to PLOS ONE policies on sharing data and materials. The SEER-Medicare data is owned by the SEER registry Principal Investigators and the Centers for Medicare and Medicaid Services. Although personal identifiers for all patient and medical care providers have been removed from the SEER-Medicare data, there remains the remote risk of re-identification (given the large amount of data available). Data can be accessed, subject to approval and data use agreement, from the Healthcare Delivery Research Program at the National Cancer Institute (*[*http://appliedresearch.cancer.gov/seermedicare/obtain/requests.html*](http://appliedresearch.cancer.gov/seermedicare/obtain/requests.html)*).”*

5. Please include captions for your Supporting Information files at the end of your manuscript, and update any in-text citations to match accordingly.

*We included captions for our Supporting Information files at the end of the manuscript (p. 24) and updated in-text citations to match:*

*S1 Appendix. Details of causal model.*

*S1 Figure. Results for discontinuation outcome.*

*S1 Table. Factor weights by cancer site and chronic condition.*
